# Transcriptome Analysis of *Zygophyllum xanthoxylum* Adaptation Strategies to Phosphate Stress

**DOI:** 10.3389/fpls.2021.723595

**Published:** 2021-10-12

**Authors:** Xiaowei Hu, Lijing Zhang, Decao Niu, Shuzhen Nan, Shujuan Wu, Hongjuan Gao, Hua Fu

**Affiliations:** State Key Laboratory of Grassland Agro-Ecosystems, Key Laboratory of Grassland Livestock Industry Innovation, Ministry of Agriculture and Rural Affairs, Engineering Research Center of Grassland Industry, Ministry of Education, College of Pastoral Agriculture Science and Technology, Lanzhou University, Lanzhou, China

**Keywords:** phosphate deficiency, phosphorus response, molecular mechanisms, transcriptome analysis, *Zygophyllum xanthoxylum*

## Abstract

Soil phosphate (Pi) deficiency is a global issue and a major constraint on plant growth. Plants typically acclimatize to low Pi by enhancing their P utilization and/or P acquisition efficiencies; however, different species have variable preferred strategies. RNA sequencing analysis was performed on the shoots and roots of *Zygophyllum xanthoxylum*, under 1 day and 10 days of Pi stress, to investigate their adaptation strategies to P deprivation. A total of 364,614 unigenes and 9,270 differentially expressed genes (DEGs) were obtained via transcriptome sequencing. An analysis of the DEGs revealed that under the 10D treatment, anthocyanin synthesis genes were upregulated under Pi stress, whereas gibberellin, ethylene, and cytokinins synthesis genes were upregulated, and abscisic acid synthesis genes were downregulated. Genes related to organic acid synthesis, encoding for purple acid phosphatases (APase) and nucleases (RNase) were upregulated under the 1D and 10D treatments, respectively. Furthermore, genes associated with Pi transport were induced by Pi stress. *Zygophyllum xanthoxylum* has special P adaptation strategies, the variation trends of genes involved in external P mobilization and acquisition, which were different from that of most other species; however, the expression levels of organophosphorus mobilization related genes, such as APases and RNases, were significantly increased. Meanwhile, PHT2s and TPTs, which distributed Pi to effective sites (e.g., chloroplast), played critical roles in the maintenance of photosynthesis. We speculated that these were economic and energy saving strategies, and there are critical adaptive mechanisms that *Z. xanthoxylum* employs to cope with deficits in Pi.

## Introduction

Phosphorus (P), which comprises an important component of nucleic acids, proteins, lipids, and cellular ATP energy, is an essential macronutrient for plant growth and development (Raghothama and Karthikeyan, 2005). On a global scale, it is estimated that ~50% of agricultural soils are deficient in P (Lynch, 2011). Sub-optimal levels of P can lead to yield losses ranging from 5 to 15% of maximal yields (Shenoy and Kalagudi, 2005). Phosphate (Pi) is the major soil fraction available for plants, which transits slowly through the soil and is easily fixed. The Pi levels of different soil types are typically not optimal for plant growth (Ye et al., 2015). More than 30% of the arable land worldwide requires supplemental P fertilization; however, excessive quantities of P fertilizer cause environmental pollution. Moreover, P fertilizer is a non-renewable resource, which is estimated to be depleted within 60 years; thus, its cost continues to rise (Vance, 2001; Vance et al., 2003). For these reasons, the selection of P efficient plants and investigations into the mechanisms involved in P efficiencies are important scientific issues that have received continuous attention in recent years (Veneklaas et al., 2012).

Plants improve their P utilization efficiency (PUE) and/or P acquisition efficiency (PAE) in response to Pi deficiencies (Manske et al., 2001; Shenoy and Kalagudi, 2005; Akhtar et al., 2008). PUE is generally improved through three strategies: (1) Extensive root systems with the capacity to explore greater soil volumes to ensure the sufficient uptake of Pi have been recognized as an important adaptation to Pi stress. Thus, Pi deficiencies increase the distribution of carbohydrates to support further growth, which leads to increased root/shoot ratios (Wissuwa et al., 2005; Chiou and Lin, 2011); (2) The production of root exudates, such as organic anions and phosphatases, may enhance the acquisition of Pi through the mobilization of insoluble sources of mineral P and organic P (Dinkelaker et al., 1989; Wang et al., 2010); (3) High-affinity Pi transporters also play an important role in Pi uptake (Dinkelaker et al., 1989; Wang et al., 2010).

The metabolic costs associated with the generation of phosphatases is unknown, where the formation of additional phosphatases is likely to comprise a small proportion of overall protein synthesis and turnover (Lynch and Ho, 2005). However, the modification of roots and exudation of organic acids typically require additional carbon inputs; thus, plants likely sacrifice carbohydrates to meet the demands required for P acquisition, which translates to a significant decline in the accumulation of biomass (Lynch and Ho, 2005; Wang et al., 2010). The PUE is primarily attributed to the efficient re-translocation and re-use of the stored P in plants. Acid phosphatase (APase) contributes to increased PUE through the remobilization of P from old leaves (Vance et al., 2003), whereas low-affinity Pi transporters are involved in vascular loading and unloading, which affects the PUE (Smith et al., 2003; Akhtar et al., 2008). Furthermore, changes in P-related metabolic pathways can facilitate the improved PUE of plants. To reduce P consumption, while ensuring sufficient supplies of P in the synthetic and metabolic pathways of important P-requiring compounds, plants can reduce non-essential P use through the replacement of compounds, or bypassing P demanding processes (Shenoy and Kalagudi, 2005; Kobayashi et al., 2006; Yang et al., 2012).

Numerous studies have revealed that the adjustment of root morphologies and physiological processes are co-existing Pi stress response strategies in plants (Vance et al., 2003; Lin et al., 2014; Zhang et al., 2014); however, differences remain between various plants. For example, maize responds to low P levels by altering its root morphologies rather than its physiological characteristics (Wen et al., 2017). *Zygophyllum xanthoxylum* is a shrub that belongs to *Zygophyllaceae*, which is endemic to the desert areas of Central Asia, and a dominant species in the deserts of Western China (Chen, 2001; Zhao and Zhu, 2003). *Zygophyllum xanthoxylum* can be consumed as a foraging plant due to the rich water and nutrient content of its leaves. Additionally, its roots may be used as traditional Chinese medicine for the treatment of digestive diseases (Zhang, 2015). Previous studies have indicated that P concentrations of both the leaves and roots, biomass, and root/shoot ratios in *Z. xanthoxylum* were not significantly altered under different soil Pi levels (Hu et al., 2020). This was not consistent with typical Pi deficiency reactions, where Pi deficiency leads to reduced P concentrations in the biomass and increased root/shoot ratios (Zhang et al., 2014).

For this study, we employed RNA sequencing (RNA-seq) to explore the response modes of Pi stress in *Z. xanthoxylum* at the transcriptional level. Based on previous results, we proposed the objective of explaining, at molecular level, how *Z. xanthoxylum* responds to Pi deficiencies by adjusting its physiological and biochemical processes rather than increasing root biomass, which is distinct from other plants.

## Materials and Methods

### Plant Materials and Experimental Treatments

*Zygophyllum xanthoxylum* seeds were collected from wild plants in Alxa League of the Inner-Mongolia Autonomous Region, in China. For the experiments we selected seeds that were uniform and full, without defects. The seeds were surface sterilized for 1 min with a 5% (v/v) bleacher and rinsed five times with sterile water, soaked in sterile water at 4°C for one day, and then germinated at 25°C in the dark for 2 days.

To determine the optimal P concentrations to be used in further experiments, we established a gradient Pi addition experiment. Seedlings of uniform size and health were transplanted into pots (10 × 10 cm), which were filled with coarse quartz sand and irrigated with a modified ½ strength Hoagland nutrient solution that contained 2 mM KNO_3_, 0.5 mM Ca(NO_3_)_2_·4H_2_O, 0.5 mM MgSO_4_·7H_2_O, 0.5 mM CaCl_2_, 50 μM H_3_BO_3_, 0.04 mM EDTA-Fe, 1.6 μM ZnSO_4_·7H_2_O, 10 μM MnCl_2_·4H_2_O, 0.05 μM Na_2_MO_4_·2H_2_O, and 0.1 μM CuSO_4_ (without P supply) for 10 days. A selection of 10-day-old seedlings were supplied with modified ½ strength Hoagland nutrient solution containing 0, 0.2, 1, 5, 50, 250, or 500 μM P for 30 days. Another set of 10-day-old seedlings were used for high P level experiments. The P supplies were subsequently increased every 2 days for a subset of the pots resulting in (after 20 days) the final three P levels, which were 1,000, 2,000, and 5,000 μM P. These seedlings were then cultured at these final high concentrations for 10 days, and the P was added as KH_2_PO_4_.

Consequently, all of the plants had grown for 40 days when the shoots (S) and roots (R) were harvested. For transcriptome analysis, uniformly sized and healthy seedlings were transplanted into pots (10 × 10 cm) filled with coarse quartz sand and irrigated with a modified ½ strength Hoagland nutrient solution (500 μM P), and the P was added as KH_2_PO_4_. The cotyledons of each plant were cut 4 weeks after planting, after which the plants were treated as follows: CP (control P level): seedlings were irrigated with modified ½ strength Hoagland nutrient solutions (500 μM P); -P: seedlings were treated with modified ½ strength Hoagland nutrient solutions without P (0 μM P).

The shoots (S) and roots (R) under each treatment were collected after the seedlings were treated for 1day (1D) and 10 days (10D), respectively. During the experiments, the solutions were renewed every 2 days and the pH of the nutrient solutions were adjusted to 5.8 ± 0.2. The seedlings were grown in a greenhouse at a temperature of 28 ± 3°C, with a photoperiod of 16 h/d (we used the artificial light with an illumination intensity of ~10,000 lx.), and relative humidity of ~30%. Following collection, a portion of the samples was oven-dried at 60°C for 96 h to achieve a constant weight for measuring the biomass and P concentrations (five biological replicates), whereas another portion was employed for RNA-Seq (three biological replicates).

### Measurement of Total Plant P Concentrations

The total plant P concentrations (% of dry mass) were measured following digestion in a di-acid mixture at an 8:1:1 ratio (HNO_3_:HClO_4_:H_2_SO_4_) employing standard methods (AOAC, 1970).

### Measurement of APase Activity and Anthocyanin Content

The shoot and root acid phosphatase (APA) activities were quantified according to the methods reported by Liu et al. (2004). The APA activity was expressed as μmol p-nitrophenol per g fresh weight per min. This experiment had five biological replicates.

Anthocyanin quantification: pre-weighed *Z. xanthoxylum* leaves were immersed in an extraction buffer (methanol-1% HCl, v/v) and maintained at 2°C for 24 h. Two absorbencies (A_532_ and A_653_) of the extracts were spectrophotometrically measured (SHIMADZU, Japan). The amount of anthocyanins was reported as (A_535_-0.24^*^A_650_) g^−1^fresh weight (FW) (Murray and Hackett, 1991). This experiment had five biological replicates.

### RNA Extraction and Assessment cDNA Preparation

The total RNA was isolated from the *Z. xanthoxylum* shoots and roots after 1D and 10D of P treatment, using TRIZOL (Invitrogen, USA). The RNA purity and concentration were determined using agarose gel electrophoresis (1.2%), Nanodrop ND-1000, Qubit 2.0, and Agilent 2100 Bioanalyze. Twenty-four qualified RNAs (CP-1D-S, -P-1D-S, CP-1D-R, -P-1D-R, CP-10D-S, -P-10D-S, CP-10D-R, -P-10D-R; including three biological replicates) were employed to create RNA sequencing libraries for the HiSeq 2500 sequencing platform.

### Illumina Transcriptome Library Preparation and Sequencing

Illumina sequencing was performed following the manufacturer's instructions. Initially, oligo(dT) beads were used for the enrichment of the eukaryotic mRNA. Subsequently, poly(A)^+^ RNA was purified and fragmented into smaller pieces. First-strand cDNA was synthesized with random hexanucleotide primers (random hexamers) using small RNA fragments as templates. Second-strand cDNA was then synthesized in a reaction mixture containing a buffer, dNTPs, RNase H, and DNA polymerase I. Subsequently, AMPure XP beads were utilized to purify the cDNA. The purified duplex cDNA was end-repaired, and poly(A) tails were added, after which AMPure XP beads were used to select fragments of particular sizes. Finally, a sequencing library was constructed via PCR enrichment. Following the construction of the library, Qubit 2.0 and an Agilent 2100 Bioanalyzer were used to determine the concentrations and sizes of the inserts. To ensure the high quality of the library, qRT-PCR was performed to measure the effective concentrations of the library reads. Next, high-throughput sequencing was conducted using an Illumina HiSeq 2500 platform with a 150 bp paired-end sequencing length.

### *De novo* Assembly and Assessment

High-quality reads were obtained through the removal of adaptor sequences and low-quality reads. Meanwhile, the quality of these clean data was estimated using the content parameters of Q30 and the GC, after which the reads were assembled into unigenes using Trinity software (Grabherr et al., 2011) with a sensitivity.

### Functional Annotation and Enrichment Analysis

The unigene sequences were aligned using BLASTX (Altschul et al., 1997) and compared with databases including Nr, Swiss-Prot, GO, KOG, and KEGG (Ashburner et al., 2000; Apweiler et al., 2004; Kanehisa et al., 2004; Koonin et al., 2004; Deng et al., 2006). The predicted amino acid sequences of the proteins encoded by the unigenes were compared to the Pfam database using HMMER software (Eddy, 1998; Bateman et al., 2004) to obtain the annotation data. The *E*-value parameters used were 1 × 10^−5^ for BLAST and 1 × 10^−10^ for HMMER.

### Quantification of Gene Expression Levels and Identification of Differentially Expressed Genes (DEGs)

Reads obtained through the sequencing of each sample were compared with the Unigene library using Bowtie (Langmead et al., 2009). According to the results, the expression levels were estimated by RSEM (Li and Dewey, 2011). The FPKM (fragments per kilobase of exon model per million mapped reads) of each gene was calculated, based on the gene length and the number of reads mapped to the gene (Trapnell et al., 2010). FPKM was employed to represent the expression abundance of the corresponding differentially expressed genes. Differential expression analysis was performed between samples using DESeq (Anders and Huber, 2010), *P* < 0.05 (Li et al., 2010), with difference |log_2_Fold Change| ≥ 0.5 as the screening criteria.

### Quantitative Reverse Transcription PCR Analysis

The RNA-seq samples were also used for quantitative reverse transcription PCR (qRT-PCR) to validate the transcriptome results. Eight genes induced by low P were selected from the shoots and roots for experimental validation. Gene expression levels were determined via qRT-PCR using the ABI QuantStudio 5 Real-Time PCR Systems (Applied Biosystems, U.S.A.) and used for TB Green-based qRT-PCR analysis (TaKaRa, China). The relative expression was normalized to the expression level of the internal control Actin (Ma et al., 2016), with the primers of selected genes listed in Supplementary Table 1. A total RNA isolation kit (Sangon, China) was employed to extract the total RNA according to the manufacturer's instructions. The first strand cDNA was obtained using a PrimeScript RT-PCR Kit (TaKaRa, China). The relative expression was calculated by the formula 2^−ΔΔCt^, and the experiments were conducted in triplicate.

### Statistical Analysis

Heatmaps were performed using MEV 4.9. The phylogenetic tree was generated using Mega 6.0 with the neighbor joining method, and bootstrap values from 1,000 replicates were indicated at each branch.

Data were presented as means ± SE based on five independent biological replicates for each sample for physiological experiments, and three independent biological replicates for RNA-seq. All statistical analyses were performed using SPSS 20 software and SigmaPlot 12.5. The data between different P supplies were analyzed with ANOVA and LSD, at *P* < 0.05; data between CP and -P treatment was assessed using a *t-*test, at *P* < 0.05.

## Results

### Determination of Optimal Growth P Concentration

**Figure 1:**
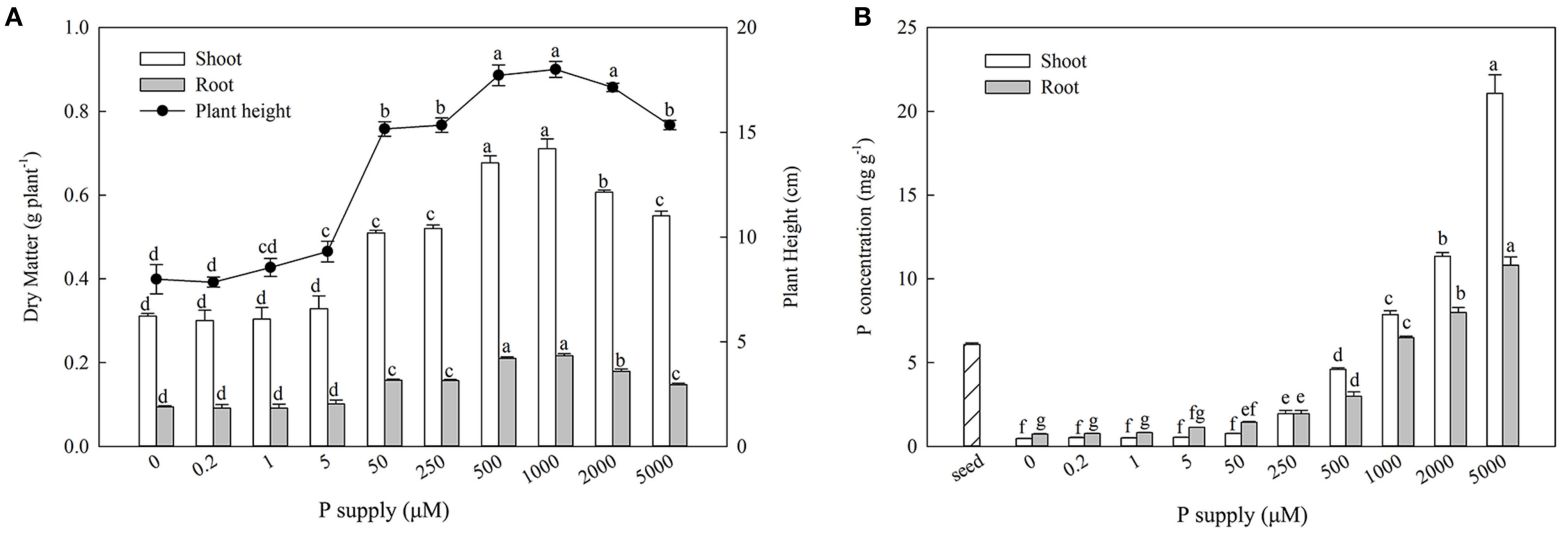
Effect of P supply on biomass, plant height **(A)**, and organ P concentrations **(B)** of *Zygophyllum xanthoxylum*. Data are means ± SE, *n* = 5; different lowercase letters above bars indicate significant differences (*P* < 0.05) between treatments.

With increasing P supplies, the shoot biomass, root biomass, and plant heights increased initially and then decreased. Maximum growth was observed at 500 and 1,000 μM Pi (Figure 1). The P concentration of the seeds was 6.08 ± 0.10 mg g^−1^, and the P concentration increased notably with P enrichment in both the shoots and roots (Figure 1).

### Phenotypic and Physiological Responses to Pi-deficiencies

From the results of the diffient P supplies (Figure 1), we decided to use 500 μM Pi as the sufficient supply of P (CP) and zero P (-P) as a deprivation treatment for an RNA-seq experiment. The P concentrations were significantly lower than those of the CP in the shoots and roots subsequent to the 1D and 10D treatments, which decreased by 12.97, 10.78%, and 24.46, 25.46%, respectively (Table 1). The P contents were also significantly lower than those of the CP in the shoots and roots following 1D and 10D treatments, which decreased by 14.06, 19.90%, and 21.81, 34.28% (*P* < 0.05), respectively. Following the 1D and 10D treatments, there were no significant differences in the biomass of the shoots and roots or root/shoot ratios (R/S) (Table 1).

**Table 1:** Effects of Pi deficiencies on P concentration, P content, biomass, and Root/Shoot ratio (R/S) in *Zygophyllum xanthoxylum*.

	**Treatment**	**P concentration (mg g** ^**−1**^ **)**		**P content (mg part** ^**−1**^ **)**		**Biomass (g plant** ^**−1**^ **)**		**R/S**
		**Shoot**	**Root**	**Shoot**	**Root**	**Shoot**	**Root**	
1D	CP	4.41 ± 0.18*	3.41 ± 0.08*	2.14 ± 0.13*	0.37 ± 0.01*	0.48 ± 0.01	0.11 ± 0.00	0.22 ± 0.01
	-P	3.76 ± 0.05	3.04 ± 0.04	1.86 ± 0.06	0.30 ± 0.03	0.50 ± 0.01	0.10 ± 0.01	0.19 ± 0.01
10D	CP	3.69 ± 0.18*	3.14 ± 0.10*	2.31 ± 0.16*	0.45 ± 0.01*	0.63 ± 0.03	0.14 ± 0.00	0.22 ± 0.01
	-P	2.79 ± 0.11	2.40 ± 0.09	1.80 ± 0.10	0.30 ± 0.02	0.64 ± 0.03	0.13 ± 0.01	0.19 ± 0.01

Data between treatments were tested with a t-test, at P < 0.05. Data are means ± SE, n = 5; Asterisks indicate significant differences (*P < 0.05) between CP and -P treatment.

### RNA-Seq

#### De Novo Assembly

In total, 184.75 Gb clean reads (Q30 ≥ 92.09%) were generated after filtering the original data, the GC content of which ranged from between 43.81 and 49.80%. All high-quality clean reads were assembled *de novo* using Trinity (Table 2), which generated 529,736 transcripts with average lengths of 742.88 nt and an N50 of 1,458 nt. Once further analyses were performed, 364,614 unigenes with mean lengths of 458.75 nt and an N50 of 528 nt were obtained.

**Table 2:** Overview of de novo sequencing and assembly.

**Length range**	**Contig**	**Transcript**	**Unigene**
200–300	44,232,435 (99.61%)	228,354 (43.11%)	206,890 (56.74%)
300–500	92,860 (0.21%)	107,562 (20.30%)	82,261 (22.56%)
500–1,000	53,624 (0.12%)	83,412 (15.75%)	47,226 (12.95%)
1,000–2,000	20,431 (0.05%)	63,026 (11.90%)	19,096 (5.24%)
2,000+	8,337 (0.02%)	47,379 (8.94%)	9,140 (2.51%)
Total number	44,407,687	529,736	364,614
Total length	1,878,569,885	393,527,930	167,265,345
N50 length	46	1,458	528
Mean length	42.30	742.88	458.75

### Identification of Differentially Expressed Genes (DEGs) Under Pi Deficiencies

**Figure 2:**
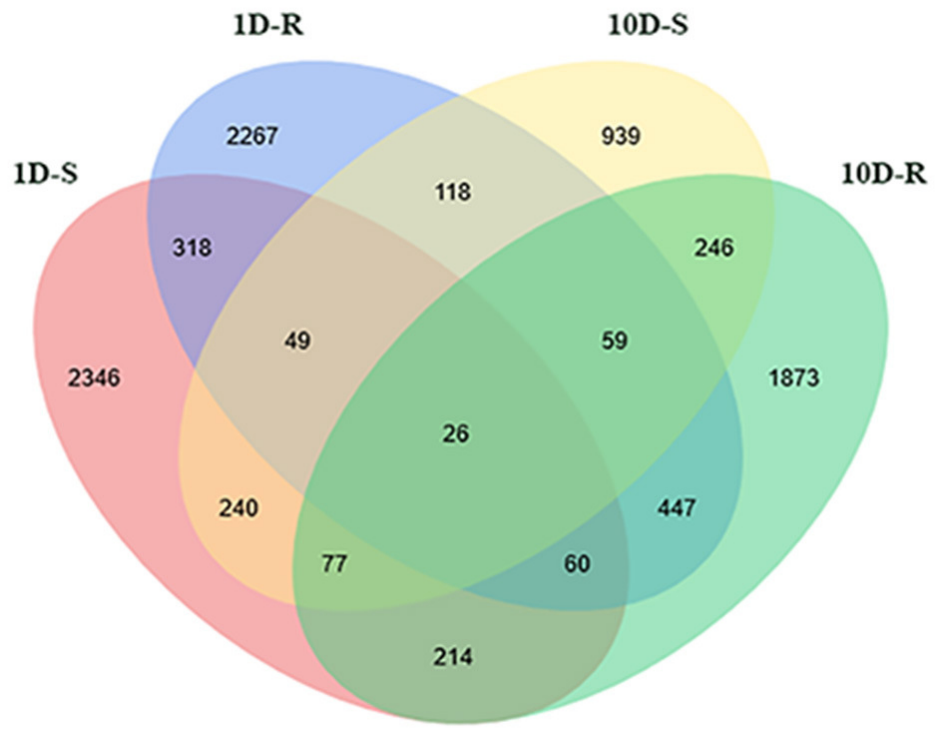
Venn diagram of DEG statistics. The labels of 1D-S, 1D-R, 10D-S, and 10D-R mean the DEGs form -P-1D-S vs. CP-1D-S, -P-1D-R vs. CP-1D-R, -P-10D-S vs. CP-10D-S, and -P-10D-R vs. CP-10D-R, respectively.

A total of 9,270 DEGs were obtained across all organ comparisons for the four treatments (Figure 2). For the 1D treatments, there were 3,330 and 3,344 DEGs in the shoots and roots, respectively. A total of 1,187 genes (35.65%) were up-regulated and 2,143 (64.35%) were down-regulated in the shoots; 1615 genes (48.30%) were up-regulated and 1,729 genes (51.70%) were down-regulated in the roots; and 453 genes (7.28%) were differentially expressed both in the shoots and roots. For the 10D treatments, there were 1,754 and 3,002 DEGs in the shoots and roots, respectively. A total of 918 genes (52.34%) were up-regulated and 836 genes (47.66%) were down-regulated in the shoots; 1,626 genes (54.16%) were up-regulated, whereas 1,376 genes (45.84%) were down-regulated in the roots (Figure 2). For the 1D and 10D treatments, there were 392 and 592 overlapping DEGs in the shoots and roots, respectively.

#### Identification of DEGs

All DEGs were aligned to the NCBI non-redundant protein (Nr), Swiss-Prot protein, Gene ontology terms (GO), euKaryotic Orthologous Groups (KOG), Protein family (Pfam), and the Kyoto Encyclopedia of Genes and Genomes (KEGG) pathway database (Table 3). Of the 9270 DEGs, 7268 DEGs (78.34%) significantly matched those deposited in the public databases.

**Table 3:** Summary of DEGs annotation (number).

**Database**	**1D-S**	**1D-R**	**10D-S**	**10D-R**	**Total**
Nr	2,374 (71.89%)	2,606 (77.93%)	1,457 (80.83%)	2,406 (80.15%)	
Swiss-Prot	1,553 (46.63%)	1,829 (54.69%)	1,023 (58.32%)	1,634 (54.43%)	
GO	1,152 (34.59%)	1,473 (44.05%)	753 (42.93%)	1,253 (41.74%)	
KOG	1,208 (36.28%)	1,343 (40.16%)	725 (41.33%)	1,263 (42.07%)	
Pfam	1,756 (52.73%)	2,221 (66.42%)	1,126 (64.20%)	1,887 (62.86%)	
KEGG	448 (13.45%)	779 (23.30%)	279 (15.91%)	556 (18.52%)	
All	2,394 (71.89%)	2,710 (81.04%)	1,464 (83.47%)	2,451 (81.65%)	7,268 (78.34%)

*The labels of 1D-S, 1D-R, 10D-S, and 10D-R mean the DEGs form -P-1D-S vs. CP-1D-S, -P-1D-R vs. CP-1D-R, -P-10D-S vs. CP-10D-S and -P-10D-R vs. CP-10D-R, respectively*.

#### Functional Classification of DEGs by GO and KEGG Pathway Analysis

**Figure 3:**
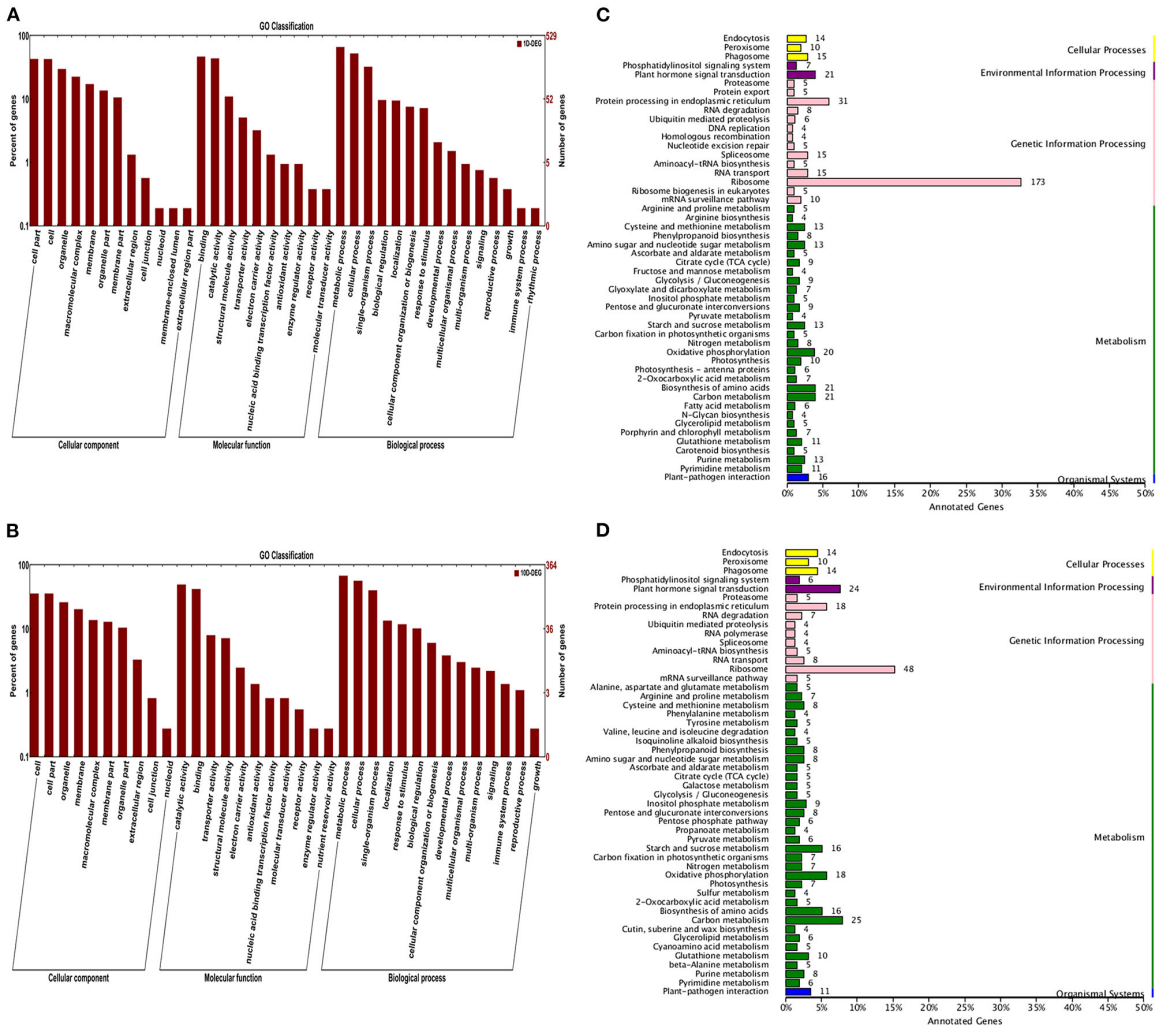
GO category and KEGG pathway enrichment analysis of the annotated DEGs. **(A,B)** Represented GO category analysis of 1D and 10D DEGs, respectively; **(C,D)** Represent the KEGG pathway enrichment analysis of 1D and 10D DEGs, respectively.

Through further analysis by GO and KEGG databases, 6,221 and 4,348 DEGs were identified in the 1D and 10D treatments, respectively. For GO analysis, the DEGs were summarized in three GO categories. Among them, the TOP enrichment terms were consistent under the 1D and 10D treatments, where in the cellular component category, “cell part” and “cell” were significantly enriched. In the molecular function category, “catalytic activity” and “binding” were significantly enriched. In the biological process category, “metabolism process” and “cellular process” were significantly enriched (Figure 3).

The KEGG pathway analysis results revealed that for the 1D treatment, the DEGs were mostly enriched in ribosome, protein processing in the endoplasmic reticulum, and plant hormone signal transduction (carbon metabolism and fatty acid metabolism). For the 10D treatment, the DEGs were primarily enriched in ribosome, carbon metabolism, and plant hormone signal transduction (Figure 3).

### Identification of DEGs Under Pi Stress

#### DEGs Related to Anthocyanin Biosynthesis

The accumulation of anthocyanin is the characteristic response of plants to Pi stress (Misson et al., 2005). In this study, Pi deficiencies initiated the significant upregulation of genes of encoding for the key enzymes in the shoots for anthocyanin synthesis (Supplementary Figure 1). For the 1D treatment, seven DEGs were up-regulated, including two *Phenylalanine ammonia lyases*, one *4-coumarate CoA ligase*, one *Chalcone synthase*, one *Flavonoid 3*′*-hydroxylase*, one *Flavonol synthase*, and one *Flavonol 3-O-glucosyl transferase*. For the 10D treatment, eight DEGs were up-regulated, including two *Phenylalanine ammonia lyases*, one *4-coumarate CoA ligase*, one *Chalcone synthase*, one *Flavonoid 3*′*-hydroxylase*, one *Flavonol synthase*, one *Dihydroflavonol-4-reductase*, and one *Flavonol 3-O-glucosyl transferase* (Table 4).

**Table 4:** DEGs involved in anthocyanin synthesis.

**Gene ID**	**Log**_**2**_ **FC**		**Annotation**
	**1D**	**10D**	
c230238.graph_c2	1.42	—	Phenylalanine ammonia lyase
c230238.graph_c1	0.91	0.79	Phenylalanine ammonia lyase
c235364.graph_c1	—	0.70	Phenylalanine ammonia lyase
c191060.graph_c0	0.86	—	4-coumarate CoA ligase
c205419.graph_c0	—	0.53	4-coumarate CoA ligase
c236325.graph_c0	1.03	0.98	Chalcone synthase
c204713.graph_c0	1.38	0.81	Flavonoid 3'-hydroxylase
c216491.graph_c0	0.68	—	Flavonol synthase
c218842.graph_c0	—	0.88	Flavonol synthase
c236317.graph_c0	—	1.49	Dihydroflavonol-4-reductase
c204713.graph_c0	1.38	0.92	Flavonol 3-O-glucosyl transferase

“*—” represented the genes were not differentially expressed*.

#### DEG Related Hormone Synthesis

**Figure 4:**
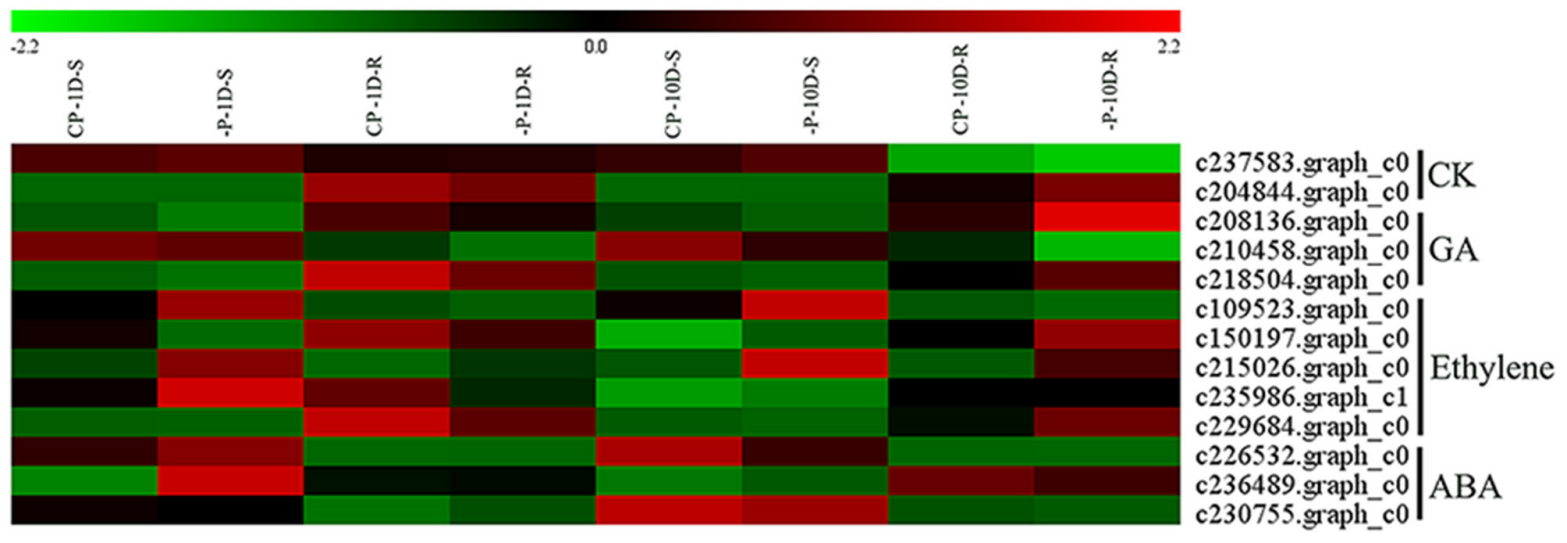
Heatmap of the DEGs involved in hormone synthesis. Expression, represented by Z scores, is shown for selected DEGs due to -P conditions. Red indicates high expression, black indicates intermediate expression, and green indicates low expression.

Phytohormones are involved in the regulation of plant growth and development under low Pi stress, which is closely related to low Pi stress (Chiou and Lin, 2011). In this study, for cytokinin (CK), there were two DEGs encoding for isopentenyltransferase (IPT) in the roots of the 10D treatment, one of which was down-regulated and the other up-regulated. For gibberellin (GA), there were one *GA*_20_*OX* and one *GA*_3_*OX* up-regulated, and one *GA*_2_*OX* down-regulated in the roots of the 10D treatment. For ethylene, under the 1D treatment, there were four DEGs in the shoots, among which two *1-aminocyclopropane-1-carboxylate oxidases* (*AOC*) and one *1-aminocyclopropane-1-carboxylate synthase* (*ACS*) were up-regulated, and one *ACS* was down-regulated. There were two DEGs in the roots, among which one *AOC* was up-regulated, and one *ACS* was down-regulated. For the 10D treatment, there was one *ACS*, two *ACO*, and two *ACS*, and one *ACO* up-regulated in the shoots and roots, respectively. For abscisic acid (ABA), under the 1D treatment, there were two *nine-cis-epoxycarotenoid dioxygenase* (*NCED*) up-regulated expressions in the shoots and roots, respectively. For the 10D treatment, there was one *NCED* downregulated expression in both the shoots and roots (Figure 4 and Supplementary Table 2).

#### DEGs Related to Organic acid Synthesis

**Figure 5:**
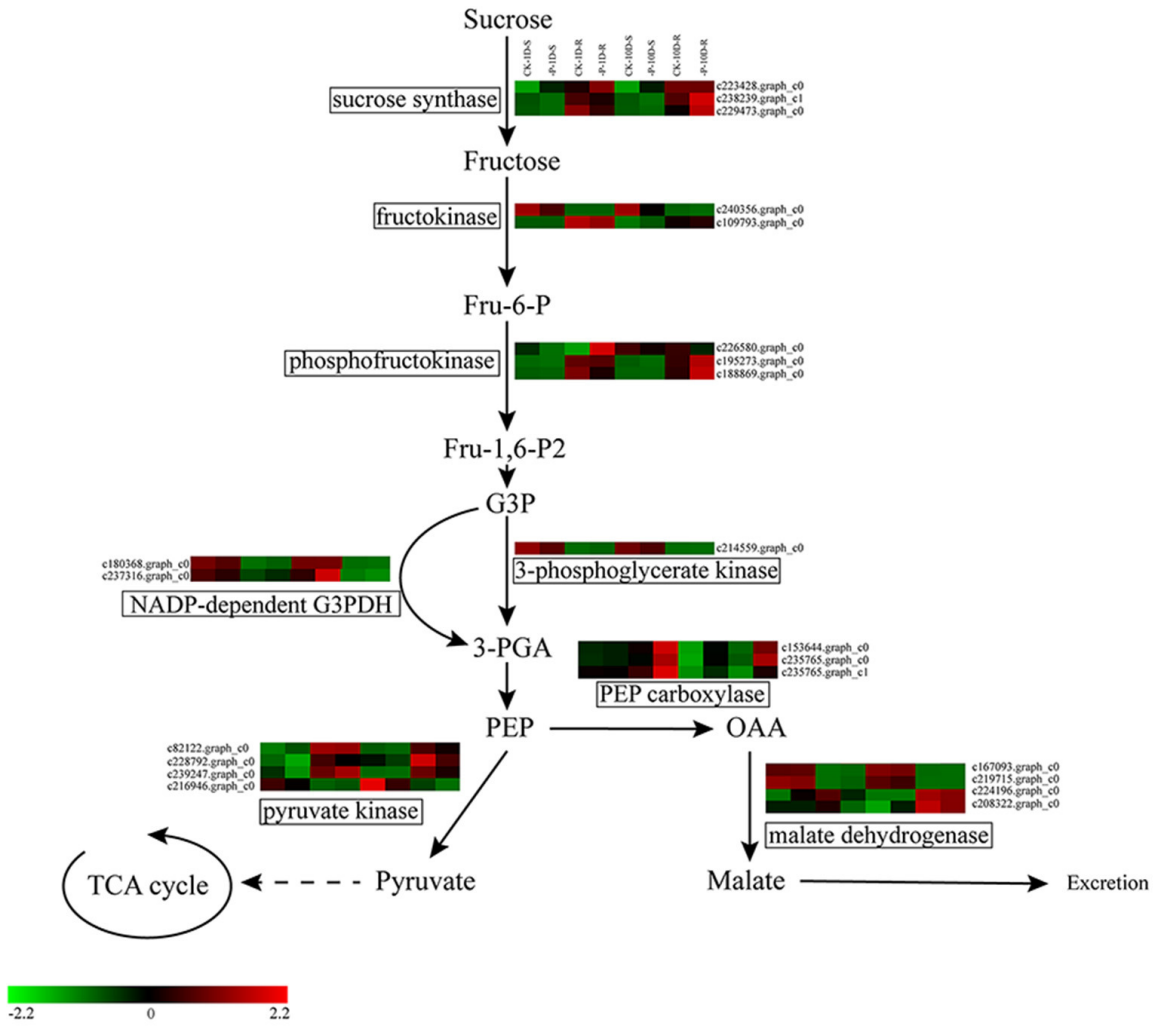
Heatmap of the DEGs involved in glycolysis and organic acid synthesis pathways. Expression, represented by Z scores, is shown for selected DEGs due to -P conditions. Red indicates high expression, black indicates intermediate expression, and green indicates low expression.

The release of organic acids is one of the essential strategies that plants employ to cope with Pi stress. In this study, 10 DEGs were up-regulated under the 1D treatment, including one *sucrose synthase*, one *phosphofructokinase*, one *NADP-dependent G3PDH*, one *3-phosphoglycerate kinase*, one *Pyruvate kinase* (*PK*), three *PEP carboxylase* (*PEPC*), and two *malate dehydrogenase* (*MDH*). Meanwhile, two *PK* and two *MDH* were down-regulated. There were 12 DEGs under the 10D treatment, among which three *sucrose synthase*, one *fructokinase*, two *phosphofructokinase*, one *NADP-dependent G3PDH*, and three *PEPC* were up-regulated, whereas one *PK* and one *fructokinase* were down-regulated (Figure 5 and Supplementary Table 3).

#### DEGs Related to Acid Phosphatases

The induction and secretion of acid phosphatase (APase) is one of the important adaptive responses of plants to low P environments (Vance et al., 2003). In this study, eight DEGs encoding for APase were identified under the 1D treatment, among which four DEGs were up-regulated. Nine DEGs encoding for APase were identified under the 10D treatment, and only one DEG was down-regulated. Of all the *APase* DEGs, two DEGs were differentially expressed both under the 1D and 10D treatments (Supplementary Table 4). Furthermore, there were 12 *purple acid phosphatase* (*PAP*) in differentially expressed *APases*. Among 12 *PAPs*, the expression level of c222117.graph_c0 was the highest, which was only upregulated under the 10D treatment, and the FPKM value increased from 32.52 to 61.13. c226904.graph_c0 was the second in rank of FPKM value, which was increased from 19.93 to 32.01 under the 10D treatment (Supplementary Table 4).

**Figure 6:**
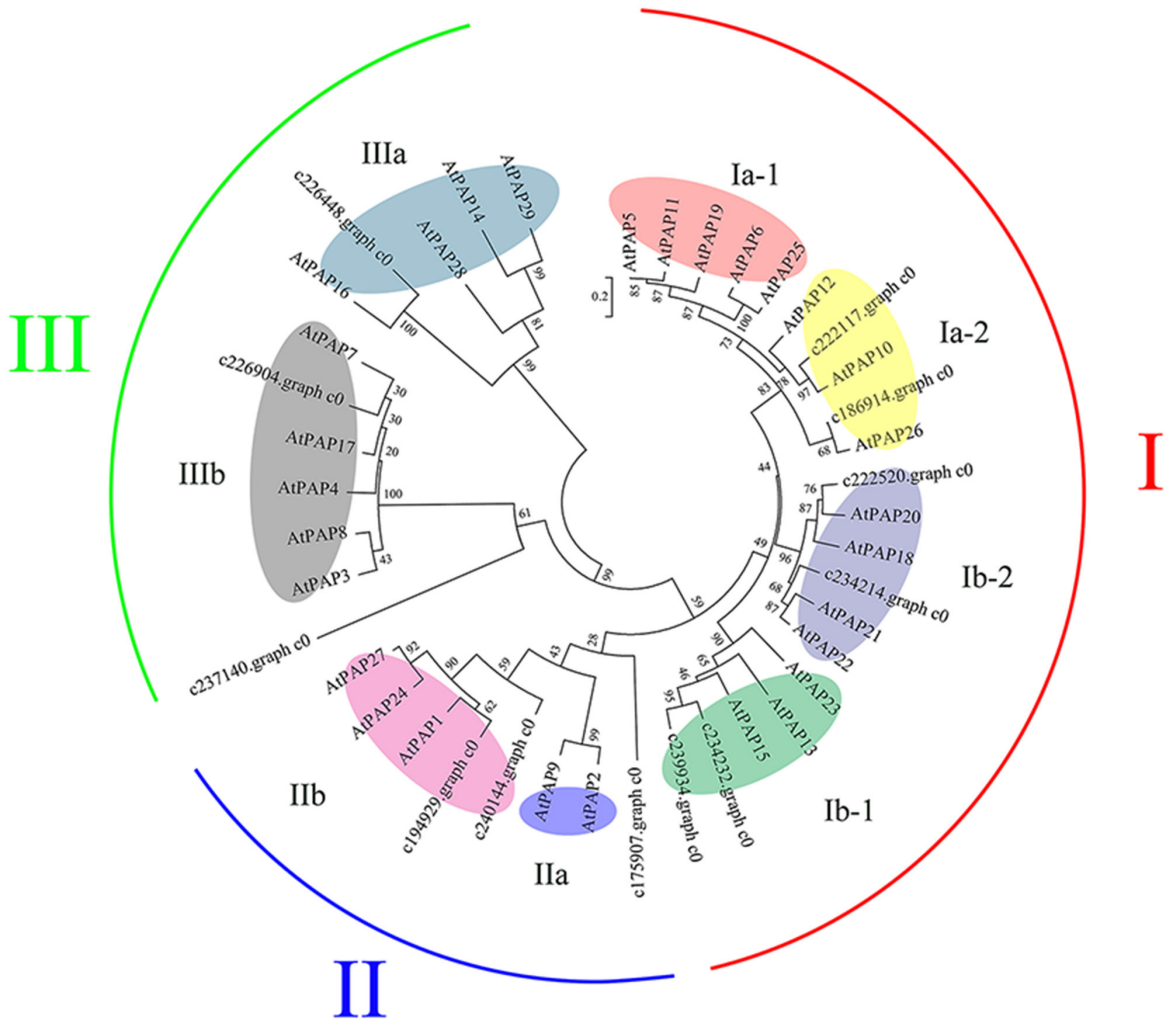
Phylogenetic tree constructed of *Zx*PAP and *At*PAP proteins. A neighbor-joining phylogenetic tree was constructed using Mega 6.0, through the multiple sequence alignment of PAP family amino acid sequences from *Arabidopsis thaliana* (*At*) and *Zygophyllum xanthoxylum* (*Zx*). Bootstrap values are percentages of 1,000 replications. The main groups (groups I, II, and III) were further divided to yield eight subgroups (colored elipses). The GenBank accession numbers of *A. thaliana* represented in the phylogenetic tree are as follows: *At*PAP1: NP_172830.1; *At*PAP2: OAP14841.1; *At*PAP3: AEE29210.1; *At*PAP4: AAW29946.1; *At*PAP5: AEE32870.2; *At*PAP6: AEE33383.1; *At*PAP7: AEC05511.1; *At*PAP8: NP_178298.2; *At*PAP9: AEC05701.1; *At*PAP10: NP_179235.1; *At*PAP11: AEC06729.1; *At*PAP12: AEC07951.1; *At*PAP13: AEC08738.1; *At*PAP14: AEC10767.1; *At*PAP15: AEE74502.1; *At*PAP16: AEE74865.1; *At*PAP17: NP_566587.1; *At*PAP18: AEE76388.1; *At*PAP19: AEE78116.1; *At*PAP20: OAP01932.1; *At*PAP21: AEE78996.1; *At*PAP22: AEE78997.1; *At*PAP23: NP_193106.3; *At*PAP24: AEE84973.1; *At*PAP25: AEE86645.1; *At*PAP26: NP_198334.1; *At*PAP27: AED95939.1; *At*PAP28: AED96852.1; *At*PAP29: AED97710.1.

The 12 *Zx*PAPs of *Z. xanthoxylum* and 29 *At*PAPs of *Arabidopsis thaliana* were analyzed via a phylogenetic tree (Figure 6), which revealed that the PAPs were divided into three groups. The I and II group proteins existed in oligomeric form when exercising functions, with more than 400 amino acid residues, whereas the III group proteins existed in the form of single molecules (Li et al., 2002). A further division of the three major groups yielded eight subgroups (Ia-1, Ia-2, Ib-1, Ib-1, IIa, IIb, IIIa, and IIIb; Figure 6).

#### DEGs Related to Ribonucleases

**Figure 7:**
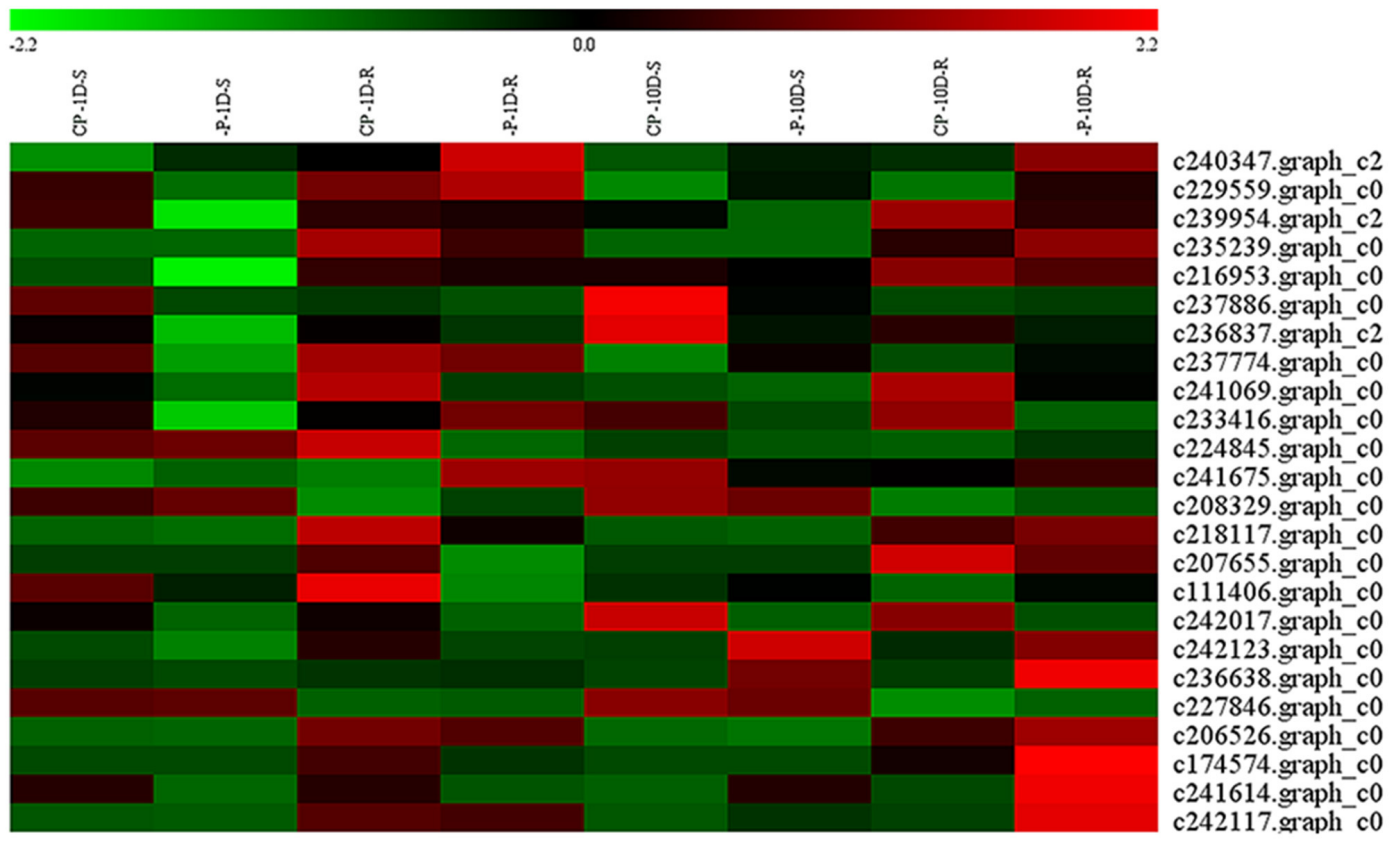
Heatmap of the DEGs encoding RNases. Expression, represented by Z scores, is shown for selected DEGs due to -P conditions. Red indicates high expression, black indicates intermediate expression, and green indicates low expression.

Ribonucleases (RNase) degraded RNA and released Pi, which allowed them to re-participate in the cycle. In this study, 24 DEGs encoding RNase were identified under Pi deficiencies. Under the 1D Pi stress, one gene was up-regulated and 10 were down-regulated in the shoots, whereas three genes were up-regulated and seven were down-regulated in the roots. Under the 10D Pi stress, three genes were up-regulated, and three genes were down-regulated in the shoots, whereas 10 genes were up-regulated and three were down-regulated in the roots (Figure 7 and Supplementary Table 5). It is worth noting that the TOP3 expression levels of *RNase*s were c235239.graph_c0, c206526.graph_c0, and c227846.graph_c0 under the 10D treatment, and the FPKM values were increased from 69.63, 28.59, and 26.23, to 118.53, 42.14, and 38.36, respectively (Supplementary Table 5).

#### DEGs Related to Phosphate Transporters

The acquisition and distribution of phosphate is mediated by phosphate transporters. In this study, seven DEGs encoding for phosphate transporters were identified under the 1D treatment, where five DEGs of these were up-regulated and annotated as two *PHO1*s, two *PHT1*s, and one PHT2-1, respectively. Under the 10D treatment, a total of seven DEGs were up-regulated and annotated as four *PHT1*s, one *PHO1*, and two *PHT2*s. The total FPKM of *PHO1*s increased from 19.89 to 21.04 under the 1D treatment. Under the 10D treatment, the expression levels of c240011.graph_c3, c175668.graph_c0, and c109288.graph_c0 were the TOP 3 expression levels, rising from 94.82, 27.55 and 23.78 to 153.66, 37.91 and 36.61, respectively (Table 5 and Supplementary Table 6), which were all annotated as *PHT1*. Further, under both the 1D and 10D treatments, there was an up-regulated DEG encoding for the triose-phosphate/phosphate translocator (TPT) (Table 5).

**Table 5:** DEGs of phosphate transporters.

**Gene ID**	Log_**2**_F		**Annotation**	**From**
	**1D**	**10D**		
c241096.graph_c0	1.7234	—	Phosphate transporter PHO1	Shoot
c234968.graph_c0	−0.6262	—	Phosphate transporter PHO1	Shoot
c216384.graph_c1	1.1778	—	Phosphate transporter PHO1	Root
c230749.graph_c0	−0.7232	—	Phosphate transporter PHO1	Root
c235756.graph_c0	—	0.6752	Phosphate transporter PHO1	Root
c240011.graph_c3	0.5336	0.7544	Inorganic phosphate transporter 1–3	Root
c109288.graph_c0	—	0.8058	Inorganic phosphate transporter 1–1	Root
c175668.graph_c0	—	0.6299	Inorganic phosphate transporter 1–4	Root
c204129.graph_c2	0.7356	—	Inorganic phosphate transporter 1–3	Root
c180900.graph_c0	—	0.9477	Inorganic phosphate transporter 1–4	Root
c219337.graph_c0	—	0.9155	Inorganic phosphate transporter 2–1	Shoot
c230324.graph_c0	2.7534	0.5026	Inorganic phosphate transporter 2–1	Shoot
c223089.graph_c0	1.1277	—	Triose phosphate/phosphate translocator	Shoot
c165074.graph_c0	—	2.0478	Triose phosphate/phosphate translocator	Shoot

“*—” represented the genes were not differentially expressed*.

### Verification Test of Physiology and qRT-PCR

#### APase Activity and Anthocyanin Content

From the above analysis, we selected two indices as physiological verification tests: APase activity and anthocyanin content. Under both the 1D and 10D treatments, the shoot and root APase activities were higher than those of the CP; however, only the roots exhibited significant differences (*P* < 0.05), which were 1.4 and 1.90 times, respectively. Under both the 1D and 10D treatments, the anthocyanin contents were higher than those of the CP, but significant differences (*P* < 0.05) were observed only under the 10D treatment, which were 1.40 times (Table 6). Changes in the APase activity and anthocyanin content were consistent with the changes in the genes, which suggested that the RNA-seq data were consistent with the physiological changes, and the analysis was reliable.

**Table 6:** Effects of Pi deficiencies on APase activity and anthocyanin content in *Zygophyllum xanthoxylum*.

	**Treatment**	**APase activity** **(μmol min**^**−1**^**g**^**−1**^ **FW)**		**Anthocyanin content** [A_**532**_-(0.24*A_**653**_) g^**−1**^ FW]
		**Shoot**	**Root**	**Shoot**
1D	CP	1.83 ± 0.09	1.43 ± 0.09	0.50 ± 0.04
	-P	2.05 ± 0.15	2.02 ± 0.06*	0.57 ± 0.02
10D	CP	1.79 ± 0.14	1.36 ± 0.02	0.52 ± 0.02
	-P	1.93 ± 0.13	2.59 ± 0.04*	0.73 ± 0.03*

Data between treatments were tested with a t-test, at P < 0.05. Data are means ± SE, n = 5; Asterisks indicate significant differences (*P < 0.05) between CP and -P treatment.

#### qRT-PCR

**Figure 8:**
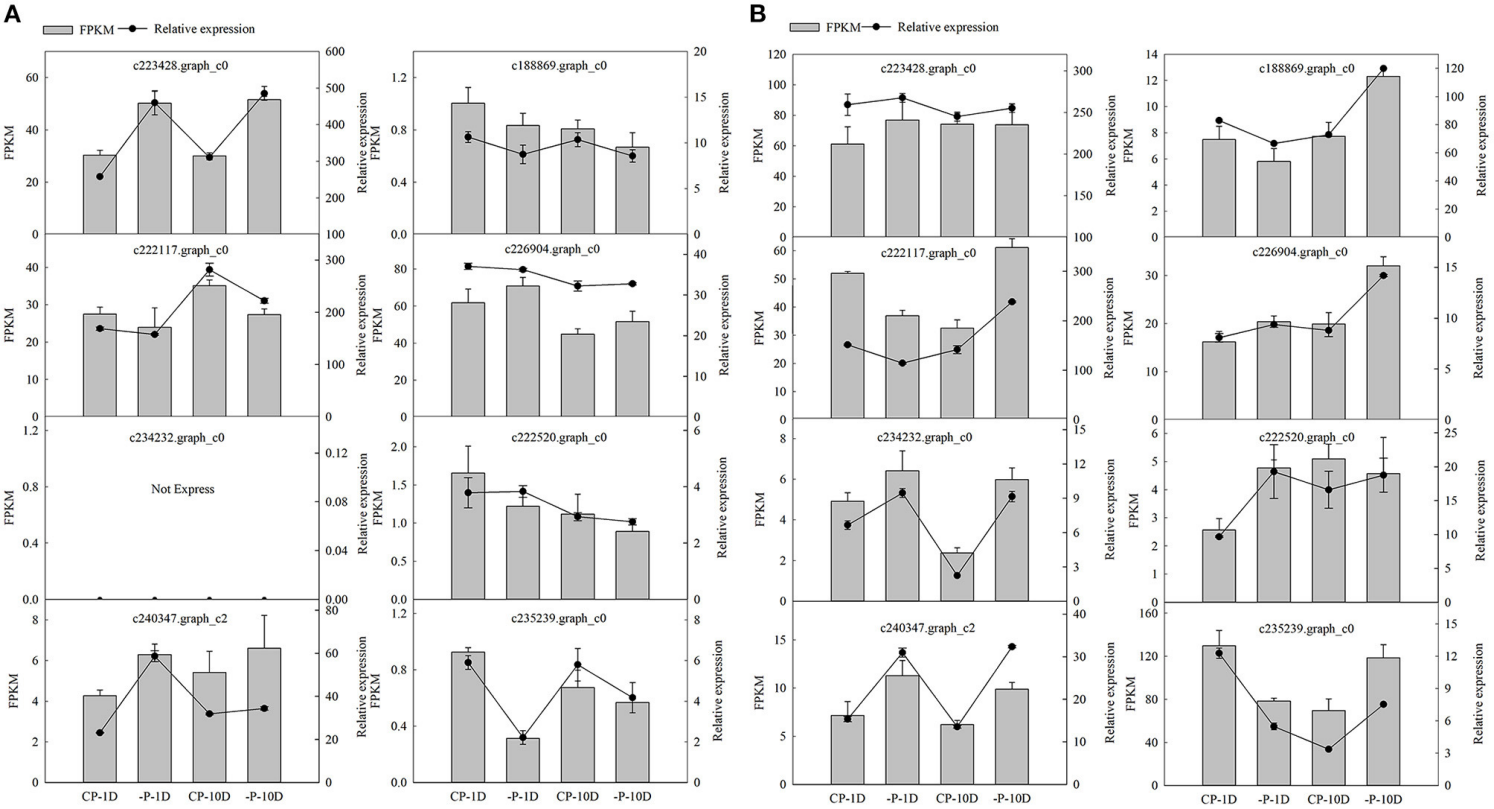
Quantitative reverse-transcription PCR validation of DEGs in shoots and roots. **(A,B)** Represent expression levels of eight DEGs based on RNA-seq and qRT-PCR in the shoots and roots, respectively. Black columns represent FPKM based on the Illumina platform. Lines represent relative expression quantity tested by qRT-PCR. The bars represent SE (*n* = 3).

Eight DEGs were selected from shoots and roots for quantitative RT-PCR analysis to validate the RNA-Seq data. The results indicated a consistent expression trend between RNA sequencing and qRT-PCR, which verified that the sequencing results were accurate, and the analysis of DEGs was reliable (Figure 8).

## Discussion

According to the results of adding different P levels, both the biomass and plant heights of *Z. xanthoxylum* attained a maximum at 500 and 1,000 μM P levels, which were significantly higher than those at 0, 0.2, 1, 5, 50, 250, 2,000, and 5,000 μM P (Figure 1). This signified that the functionalities of the plants were inhibited by P deficiencies or excesses. Thus, for *Z. xanthoxylum*, an optimal P level of 500 μM P was observed to maintain maximum biomass, which was selected as the control group (CP) for transcriptome sequencing.

A decrease in organ P concentrations is one of the factors that determines the responses of plants to P (Byrne et al., 2011). In this study, compared with CP, the P concentrations of the roots and shoots under the 1D and 10D treatments were significantly decreased (Table 1). The accumulation of anthocyanin is a characteristic response of plants to Pi deficiencies (Stewart et al., 2001; Misson et al., 2005). The genes coding for key enzymes involved in anthocyanin synthesis (Misson et al., 2005) were significantly up-regulated under the 1D and 10D Pi stresses (Table 4), where the anthocyanin content increased under the 10D Pi stress (Table 6).

The results above suggested that our experimental conditions successfully reproduced an effect on the Pi levels and that the time frame (1D) was sufficient to elicit a Pi stress response. However, no significant differences in the biomass of *Z. xanthoxylum* were observed (Table 1), which indicated that growth was not restricted, which was consistent with the conclusion that *Z. xanthoxylum* possessed high PUE (Hu et al., 2020).

### *Zygophyllum xanthoxylum* Does Not Sacrifice Carbohydrates to Obtain More P Resources

Abundant research has shown that the reconstruction of root structures and adjustment of physiological processes are common responses of plants to Pi deficiencies (Byrne et al., 2011; Peret et al., 2011; Zhang et al., 2014); however, these adaptation strategies vary by species. For example, maize responds to Pi deficiencies by altering its root morphologies rather than increasing root exudations (Wen et al., 2017). Plant hormones play a critical role in the remodeling of root morphologies (Chiou and Lin, 2011). Pi defiencies typically result in the accumulation of auxin and ethylene, wheras GA, CKs, and ABA are decreased, which inhibits the growth of primary roots while promoting the formation of lateral roots and hairs (Zhang et al., 2014).

In this study, the key genes involved in auxin synthesis were not significantly altered under long-term treatments; however, GA, ethylene, and CK synthesis genes were up-regulated, and ABA synthesis genes were down-regulated (Figure 4). From RNA-seq data, we speculated that the changes in the contents of ethylene and ABA in *Z. xanthoxylum* were consistent with other plants; however, the changes in GA, CKs, and auxin were different from other plants. Thus, we hypothesized that differ hormonal changes might be a key explanation for the absence of significant changes in the root biomass and R/S ratios (Table 1). In this study, the unchanged R/S ratios of *Z. xanthoxylum* indicated that it did not obtain additional P resources through increased root biomass.

Soil resident inorganic P is generally chemically bound, which only becomes available to plants when solubilized by H^+^ or organic anions (Zhang et al., 2014). Under Pi deficiency conditions, plants release the salts of organic acids from roots into the soil as an important survival strategy (Ligaba et al., 2004; Liao et al., 2006; Jemo et al., 2007; Cheng et al., 2011). In this investigation, key genes involved in the glycolysis pathway increased their expression under Pi deficiencies (Figure 5 and Supplementary Table 3). Under the 1D and 10D treatments, the *PEPC*s were up-regulated, which indicated that more substrates were involved in the TCA cycle. This was consistent with the view that the up-regulated expression of genes in the glycolytic pathway provides sufficient carbon sources for the TCA cycle under Pi deficiency conditions (Li et al., 2010; Zhang et al., 2014). Meanwhile, the *MDH*s were up-regulated under the 1D treatment.

Increased MDH activities can significantly enhance the root exudation of malate (Lu et al., 2012); however, it can also increase the exudation of citric acid, oxalic acid, succinate, and acetic acid (Tesfaye et al., 2001). For the 10D treatment, no gene upregulation related to organic acid synthesis was found. From the above results, at the molecular level, we speculated that Pi deficiencies might result in enhanced glycolysis to provide additional carbon sources for organic acid synthesis; however, the secretion of organic acids might be a short-term response rather than a long-term adaptation strategy to compensate for Pi deficiencies.

The restriction of plant growth is one of the major limitations caused by Pi deficiencies, which may be due to the fact that the modification of root systems and the exudation of organic acids require additional carbon inputs; thus, plants need to sacrifice carbohydrates to obtain more P nutrients (Wang et al., 2010). In our study, the expression of key genes involved in the synthesis of hormones and organic acids were not altered. Thus, we speculated that *Z. xanthoxylum* did not utilize additional carbon for the larger development of roots, or more organic acids were synthesized, which was an important factor for the stability of biomass. The design of relative physiological experiments will be required in the future to prove this inference.

### Remobilization of Organic P

Acid phosphatases (APases) catalyze the hydrolysis of Pi from a broad range of P-monoesters and anhydrides with an acidic pH optimum (Tran et al., 2010a). Low P-induced APase exists in two forms, including intracellular APase, which releases Pi from vacuoles and remobilizes P from old leaves to facilitate its recycling in plants (Vance et al., 2003; Robinson et al., 2012). The second form is exuded APase, which is secreted at the root surface into the surrounding environment and releases Pi from soil residing organic P (Ticconi and Abel, 2004; Fang et al., 2009).

Under the 1D treatment in this study, eight DEGs encoding for APase were identified, among which four DEGs were up-regulated. Under the 10D treatment, nine DEGs encoding for APase were identified, and only one DEG was down-regulated. Among the 15 *APase* above, 12 were *purple acid phosphatase*s (PAP). This was consistent with the conclusion that the APase closely related to low P stress was primarily PAP. It was discovered that the expression levels of *PAP*s were induced and increased under low P conditions across many species (Tran et al., 2010b).

An analysis of the evolutionary tree revealed that a total of 12 *Zx*PAPs belonged to the I(6), II(3), and III (3) groups (Figure 6). *At*PAP10, *At*PAP12, and *At*PAP26 play crucial roles in the decomposition of organic P *in vitro*, as well as the recovery of P *in vivo* (Hurley et al., 2010; Tran et al., 2010b). *At*PAP10 is predominantly associated with the root surface following secretion, and accounts for 30% of the total PAP activity (Wang et al., 2014). *At*PAP12 is the major PAP secreted into the rhizosphere (Zhu et al., 2005; Tran et al., 2010b), whereas *At*PAP26 is dually targeted to the cell vacuole and secretome (Zhu et al., 2005; Tran et al., 2010b). The c222117.graph_c0 and c186914.graph_c0 with the above three *At*PAPs all belonged to the subgroup Ia-2. Due to the expression level of c222117.graph_c0, it was the highest between the 12 *ZxPAP*s (Supplementary Table 4). We speculated that it played a vital role in the remobilization of internal and external P during long-term Pi deficiencies. The expression levels of c222520.graph_c0 and c234214.graph_c0 were augmented under the 1D and 10D treatments, respectively, which together with *At*PAP18 belonged to subgroup Ib-2.

AtPAP18 is a dual-targeted protein, which confers the efficient retrieval of Pi from bound extracellular compounds, as well as expendable intracellular Pi-monoesters and anhydrides (Zamani et al., 2012). We speculated that these two genes had similar functions to *At*PAP18 and belonged to double-targeted proteins, which played roles in the mobilization of internal and external P. c234232.graph_c0 and c239934.graph_c0 and were located in subgroup Ib-1. In this subgroup, both *At*PAP15 and *At*PAP23 exhibited phytase activity, where phytic acids are crucial for the storage of P in plants (Zhu et al., 2005; Kuang et al., 2009). *At*PAP15 is primarily expressed in vascular tissues, pollen grains, and roots, but not in the root tips and hairs, whereas *At*PAP23 is expressed only in flowers, and may only play a role in the utilization of endogenous P (Zhu et al., 2005; Kuang et al., 2009). It was worth noting that c234232.graph_c0 was up-regulated under the 10D treatment; thus, we speculated that c234232.graph_c0 possessed phytase activity, which primarily relied on this gene to utilize the phytase pool.

The II group contained three *Zx*PAPs, among which c240144.graph_c0 and c194929.graph_c0 belonged to subgroup IIa, whereas in the subgroup, only *At*PAP1 was reported to have a relationship with the accumulation of anthocyanin (Chhon et al., 2020) Another *Zx*PAP formed an independent branch in the II group; however, its expression was down-regulated under Pi deficiencies. It may be inferred from the above results that none of the three *Zx*PAPs may have been directly involved in the mobilization of P.

*At*PAP7, and *At*PAP17 with c226904. graph_c0 were in the same subgroup of the III group. *At*PAP7 and *At*PAP17 are both located in the endoplasmic reticulum and peroxidase, and exhibit peroxidase activity in addition to phosphatase activity. Consequently, these PAPs play roles in P remobilization and ROS metabolism (Del Pozo et al., 1999; Kataya et al., 2016). Due to the expression level rank of c226904.graph_c0, it was second among the 12 *ZxPAP*s; thus, we speculated that it was not only significantly beneficial for the reuse of P *in vivo*, but also alleviated oxidative damage.

In order of size, P pools are typically RNA > P-lipid > P-ester > DNA > metabolically active Pi. The nucleic acid pool accounts 40–60% of the P found in the combined organic P pool, and contains at least 85% RNA (Veneklaas et al., 2012), where RNase can degrade RNA and release Pi (Shimizu et al., 2001). Under Pi stress, the intracellular and extracellular RNase activity of *A. thaliana* increased significantly, which signified that RNA was an important facilitator for P remobilization (Veneklaas et al., 2012). In this study, under 1D of Pi stress, the encoding of RNase was up-regulated, and 10 encoding RNases were down-regulated in the shoots, whereas three genes were up-regulated, and seven genes were down-regulated in the roots.

Under 10D of Pi stress, three genes were up-regulated, and three genes were down-regulated in shoots, whereas nine genes were up-regulated and three genes were down-regulated in the roots (Figure 7 and Supplementary Table 5). It could be seen that with the increased duration of stress, *Z. xanthoxylum* enhanced the remobilization of the RNA pool. The above results indicated that *Z. xanthoxylum* did not use an RNA pool as a P source during short-term Pi deficiencies. However, under prolonged treatments, the RNA pool was mobilized, which enhanced the PUE. c235239.graph_c0, c206526.graph_c0, and c227846.graph_c0 might play key roles in the remobilization of the RNA pool, due to the high FPKM of the three genes under the 10D treatment.

### Pi Transporters

Pi transport is primarily accomplished via Pi transporters (Wang et al., 2017), which can be distinguished as plasma membrane transporters (PHT1 and PHO1) and intracellular transporters (PHT2, PHT3, PHT4, and PHT5; Mlodzinska and Zboinska, 2016). PHT1 and PHO1, are responsible for Pi uptake from the soil and its further allocation to aboveground plant organs and between plant tissues (Mlodzinska and Zboinska, 2016). In this study, *PHT1*s and *PHO1*s were significantly up-regulated in the roots. Two *PHT1* genes were up-regulated under the 1D treatment, as were four *PHT1* genes under the 10D treatment. These results suggested that the up-regulated expression of *PHO1*s and *PHT1*s caused by Pi deficiencies not only enhanced the absorption of external Pi, but also augmented Pi transport to improve the PUE. PHT2s are located in the inner envelope membranes of chloroplasts, whereas low affinity Pi transporters, they mediate the transport of Pi from the cytoplasm to chloroplast, while participating in the redistribution of Pi between old and young leaves (Versaw and Harrison, 2002).

In this study, the expression levels of two *PHT2*s were up-regulated in shoots under the 10D treatment, which was consistent with the conclusion that the *PHT2*s are predominantly expressed in green tissues (Versaw and Harrison, 2002). This was also consistent with the hypothesis that *Z. xanthoxylum* primarily employs internal P pools as the means to deal with long-term Pi deficiencies. The triose-phosphate/phosphate translocator (TPT) can transport photosynthetically fixed carbon from the chloroplast to cytosol, and transport Pi from the cytosol to the chloroplast stroma (Lee et al., 2017). In this study, under both 1D and 10D treatments, there was an up-regulated DEG encoding for the triose-phosphate/phosphate translocator (TPT; Table 5). We speculated that the up-regulated TPTs might be a key factor behind why Pi deficiencies did not cause biomass reduction in *Z. xanthoxylum*. The up-regulated TPTs ensured the smooth output of photosynthetic products, and enabled Pi in the cytoplasm to continuously enter chloroplasts to maintain stable photosynthesis.

### Construction of a Putative Model

**Figure 9:**
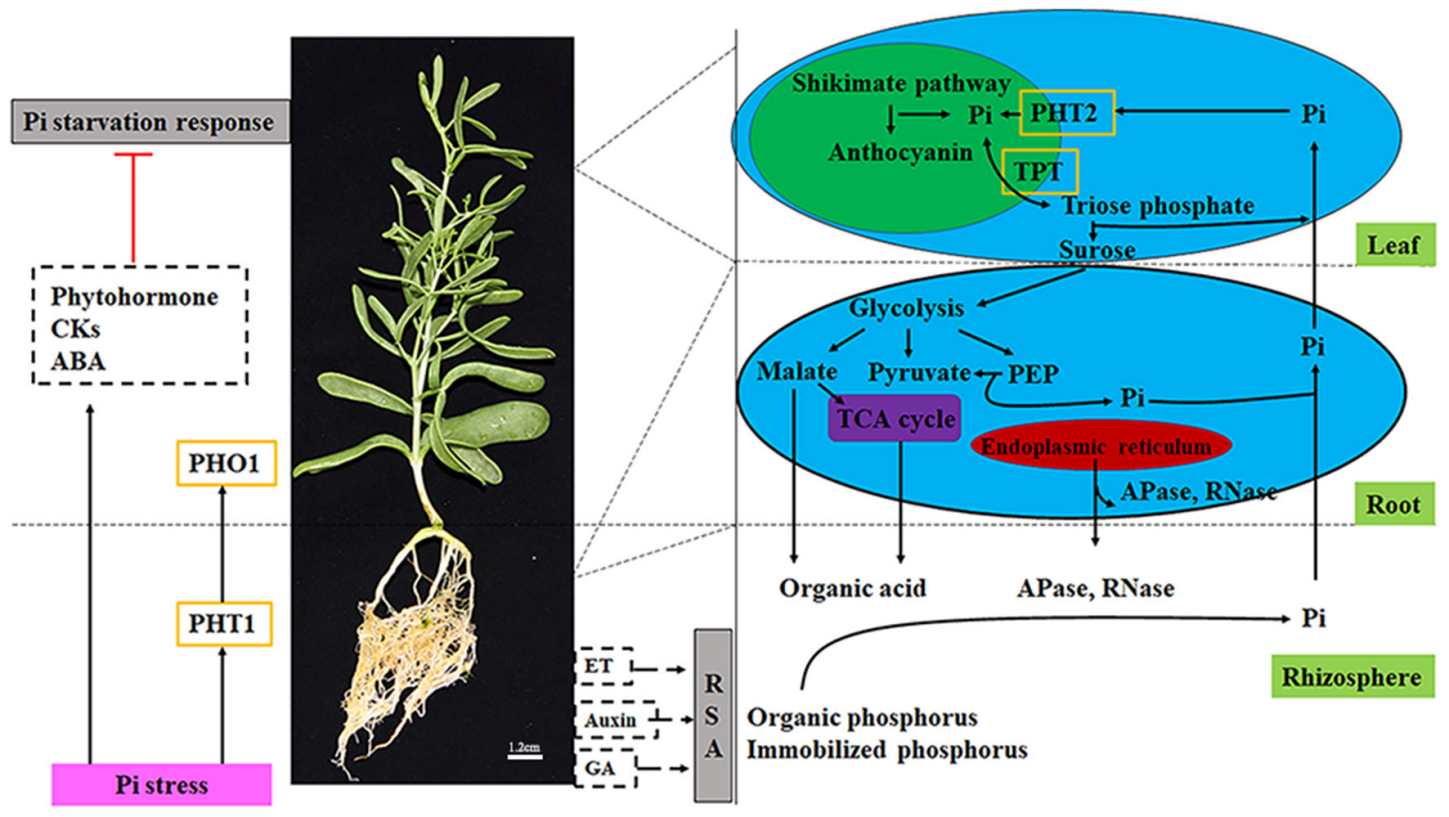
Illustration of key components involved in the Pi stress metabolic pathway in *Zygophyllum xanthoxylum*. Black solid arrows and red terminated lines represent positive regulation and suppressed expression, respectively. Dotted black arrows indicate no-effect. Gray boxes represent responses of Pi deficiency. Pink boxes show the condition of the external environment. Dotted boxes represent plant hormones. Words with green backgrounds express plant and rhizosphere tissues. Blue ovals show the cells in the roots and leaves. Within cells, green ovals represent the chloroplast. Purple ovals represent the mitochondria. Red ovals represent the endoplasmic reticulum. Yellow boxes represent transporters. CK, cytokinins; GA, gibberellin; ABA, abscisic acid; ET, ethylene; RSA, root system architecture; Pi, inorganic phosphorus; PEP, phosphoenolpyruvate; TCA cycle, tricarboxylic acid cycle. Redrawn from Ren et al. (2018).

We constructed a theoretical model based on pivotal data at the transcriptional level (Figure 9). The sensing of P limitations is the initial step in responding to changes in P nutrients. In *Z. xanthoxylum*, Pi transporters (*PHT1*s and *PHO1*s) are involved in Pi absorption and transport. Simultaneously, plant hormones (CKs and ABA) are involved in signal transduction under Pi deficiencies. The transcriptional analysis of GA, CKs, and auxin related genes implied that *Z. xanthoxylum* might not increase root biomass to forage for additional P resources. Further, RNA-seq data also implied that short-term Pi deficiencies might prompt increases in organic acid metabolism; however, increases in APases and RNases might be primarily involved in longer strategies toward the improvement of P efficiencies. As a low affinity Pi transporter, PHT2s are involved in the redistribution of Pi between old and young leaves. TPTs are engaged in the output of photosynthetic products and the transport of Pi to chloroplasts to meet the requirements for photosynthesis. The above model was proposed based on transcriptome data. In the design of future experiments, we will refine the model at the physiological level through direct physiological measurements.

## Conclusion

The *Z. xanthoxylum* response modes to P stress are special. The variable trends of genes involved in external P mobilization and acquisition, such as syntheses of organic acid and hormones, were different from that of most other species. However, the expression levels of organophosphorus mobilization related genes, such as APases and RNases, were significantly increased. Meanwhile, PHT2s and TPTs, which could distribute Pi to effective plant sites (e.g., chloroplast), played critical roles in maintenance of photosynthesis. These distinct responses might serve as important adaptive mechanisms for *Z. xanthoxylum* to cope with Pi deficiencies. We speculated that *Z. xanthoxylum* possessed the P adaptation strategy of “economy and energy saving,” and might not obtain additional P at the expense of carbohydrates. Rather, it primarily depended on the remobilization and redistribution of P to improve its efficiencies. This study provides a new perspective for improving crop yields.

## Data Availability Statement

The original contributions presented in the study are publicly available. This data can be found here: National Center for Biotechnology Information (NCBI) BioProject database under accession number PRJNA684791.

## Author Contributions

LZ and XH conceived and designed the experiments. XH, HG, and SN performed the experiments and analyzed the data. XH wrote the manuscript. LZ, HF, DN, and SW provided editorial advice. All authors contributed to the article and approved the submitted version.

## Funding

This study was funded by the National Natural Science Foundation of China (31770763); the National Key R&D Program of China (2016YFC0500506); the National Basic Research Program of China (2014CB138703); the Fundamental Research Funds for the Central Universities (lzujbky-2017-54); the Changjiang Scholars and Innovative Research Team in University (IRT_17R50), and the 111 project (B12002).

## Conflict of Interest

The authors declare that the research was conducted in the absence of any commercial or financial relationships that could be construed as a potential conflict of interest.

## Publisher's Note

All claims expressed in this article are solely those of the authors and do not necessarily represent those of their affiliated organizations, or those of the publisher, the editors and the reviewers. Any product that may be evaluated in this article, or claim that may be made by its manufacturer, is not guaranteed or endorsed by the publisher.
