# Supplementary material for: Transcriptome Analysis of Zygophyllum xanthoxylum Adaptation Strategies to Phosphate Stress
Source: Front Plant Sci. 2021 Oct 12;12:723595. doi: 10.3389/fpls.2021.723595 (PMC8545990; doi:10.3389/fpls.2021.723595)
Supplement: Supplementary file 7 [file Data_Sheet_1.docx]

Figure S1. The keys genes involved in anthocyanin biosynthetic pathway.


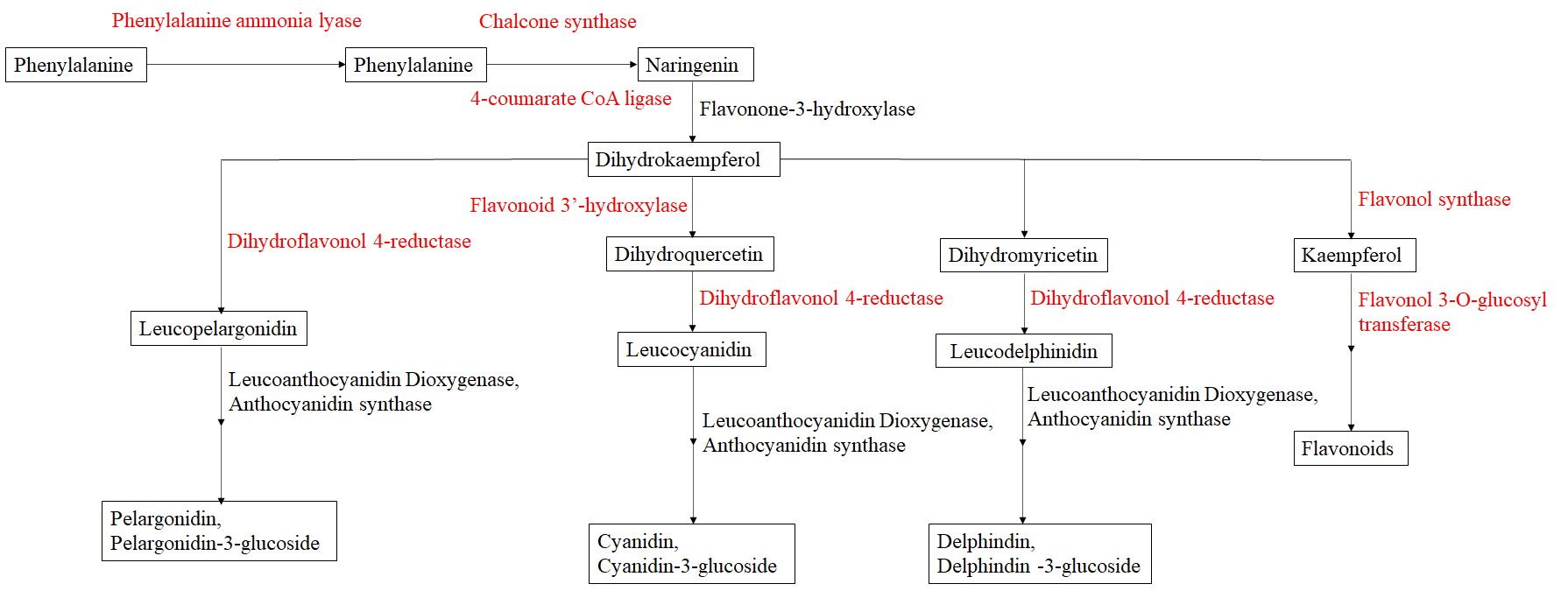


Note: The DEGs are marked in red font. The pathway redrew from Misson et al. (2005).

Misson, J., Raghothama, K.G., Jain, A., Jouhet, J., Block, M.A., Bligny, R., et al. (2005). A genome-wide transcriptional analysis using *Arabidopsis thaliana* Affymetrix gene chips determined plant responses to phosphate deprivation. *Proc. Natl. Acad. Sci. U. S. A.* 102**,** 11934-11939. doi:10.1073/pnas.0505266102.
